# Administration of follicle-stimulating hormone induces autophagy via upregulation of HIF-1*α* in mouse granulosa cells

**DOI:** 10.1038/cddis.2017.371

**Published:** 2017-08-17

**Authors:** Jilong Zhou, Wang Yao, Chengyu Li, Wangjun Wu, Qifa Li, Honglin Liu

**Affiliations:** 1 College of Animal Science and Technology, Nanjing Agricultural University, Nanjing 210095, China

## Abstract

Recent studies reported the important role of autophagy in follicular development. However, the underlying molecular mechanisms remain elusive. In this study, we investigated the effect of follicle-stimulating hormone (FSH) on mouse granulosa cells (MGCs). Results indicated that autophagy was induced by FSH, which is known to be the dominant hormone regulating follicular development and granulosa cell (GC) proliferation. The activation of mammalian target of rapamycin (mTOR), a master regulator of autophagy, was inhibited during the process of MGC autophagy. Moreover, MHY1485 (an agonist of mTOR) significantly suppressed autophagy signaling by activating mTOR. The expression of hypoxia-inducible factor 1-alpha (HIF-1*α*) was increased after FSH treatment. Blocking hypoxia-inducible factor 1-alpha attenuated autophagy signaling. *In vitro*, CoCl_2_-induced hypoxia enhanced cell autophagy and affected the expression of beclin1 and BCL2/adenovirus E1B interacting protein 3 (Bnip3) in the presence of FSH. Knockdown of beclin1 and Bnip3 suppressed autophagy signaling in MGCs. Furthermore, our *in vivo* study demonstrated that the FSH-induced increase in weight was significantly reduced after effectively inhibiting autophagy with chloroquine, which was correlated with incomplete mitophagy process through the PINK1-Parkin pathway, delayed cell cycle, and reduced cell proliferation rate. In addition, chloroquine treatment decreased inhibin alpha subunit, but enhanced the expression of 3 beta-hydroxysteroid dehydrogenase. Blocking autophagy resulted in a significantly lower percentage of antral and preovulatory follicles after FSH stimulation. In conclusion, our results indicate that FSH induces autophagy signaling in MGCs via HIF-1*α*. In addition, our results provide evidence that autophagy induced by FSH is related to follicle development and atresia.

Macroautophagy (hereafter referred to as autophagy) is a constitutive, dynamic, evolutionarily conserved catabolic process that involves the degradation and recycling of cellular constituents.^1, 2^ It maintains cell survival under various forms of stress conditions such as starvation,^3^ hypoxia,^4^ and interruption of growth signaling.^5^ Autophagy is involved in many physiological and developmental processes such as cell survival, cell death, metabolism, and innate immunity.^6, 7, 8^ Abnormalities in autophagy are implicated in numerous human diseases, including developmental disorders, neurodegenerative diseases, and cancer.^9^

A granulosa cell or follicular cell is a somatic cell of the sex cord that is closely associated with the developing female gamete (called an oocyte or egg) in the mammalian ovary. Ovarian follicle atresia is a periodic process by which immature ovarian follicles degenerate and are reabsorbed during the follicular phase of the ovarian cycle. This complicated, dynamic process is triggered by GC apoptosis.^10, 11^ Recently, increasing evidence shows that autophagy plays a vital role in survival and proliferation of GCs.^12, 13^ In rat GCs, accumulation of autophagosomes induced by serum starvation activates cell apoptosis through decreased Bcl-2 expression and, subsequently, leads to caspase activation,^14^ which suggests that a certain degree of autophagy may promote GC apoptosis. In a mouse model, inhibition of autophagy in IL-33(-/-) mice leads to massive accumulation of tissue waste containing aging-related catabolic waste during folliculogenesis, indicating a role for autophagy in removing unnecessary cells and maintaining the metabolic balance in antral follicles.^15^ Interestingly, specific knockout of autophagy-related genes results in a significant decrease in primordial follicle pools,^16, 17^ which suggests an important role for autophagy in the regulation of follicular development and maintenance of the ovarian primordial follicle reserve.

Factors related to follicular development and GC proliferation have been extensively investigated, including death ligands and receptors, caspases, Bcl-2 family members, and gonadotropins.^18^ Follicle-stimulating hormone (FSH) is synthesized and secreted by the gonadotropic cells of the anterior pituitary gland and plays an important role in follicular development, GC proliferation, and reproductive processes. Exogenous FSH is widely used to stimulate the development of mature follicles because of its effectiveness in preventing GC apoptosis and follicle atresia. Research indicated that almost all gonadotropins can significantly inhibit GC apoptosis and follicle atresia, with FSH exhibiting the highest efficiency.^19, 20^ Exposing immature rats to eCG can significantly decreased autophagy signaling at days 1 and 2, but increased it at day 3 and maintained it until day 5,^21^ suggesting a complex regulation of autophagy during follicular development. However, the relationship between autophagy and inhibition of cell apoptosis and follicle atresia by FSH remains unclear. In this study, we investigated the molecular regulation of autophagy in FSH-treated MGCs to determine the role of FSH in GC autophagy and apoptosis.

## Results

### FSH promotes MGC autophagy *in vivo*

**Figure 1:**
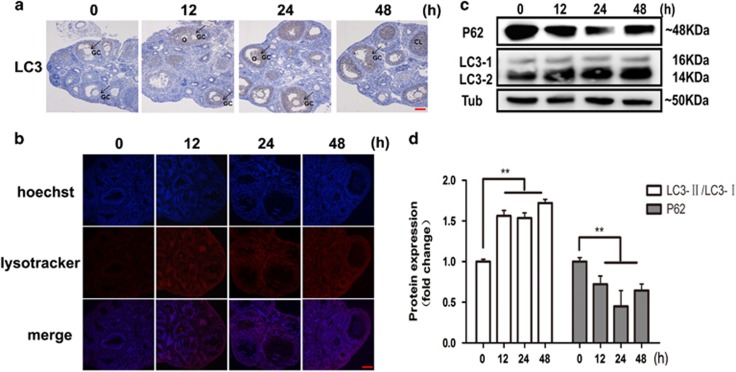
FSH induces MGC autophagy *in vivo*. (**a**) Mice were intraperitoneally injected with FSH. LC3 expression of follicular MGCs in the ovary sections was increased after FSH injection. Ovary sections were immunostained with anti-LC3 as described in Materials and Methods section, and autophagy was assessed at 0, 12, 24, and 48 h. Bar=100 *μ*m. *O*, oocyte; *GC*, granulosa cells; *CL*, corpora luteum. (**b**) FSH increased lysotracker red staining in MGCs. Lysotracker red staining (red) and DAPI (blue) was performed after treatment. Bar=100 *μ*m (**c**) FSH increased the conversion of LC3-I into LC3-II and decreased the p62 protein level in MGCs. Western blot results of extracts from cells treated with FSH (*n*=3). *α*-Tubulin was used as a loading control. (**d**) Quantitative analysis of the data presented in **c** (mean±S.E of independent experiments, *n*=3, **P<0.01)

To determine whether the function of FSH is correlated with autophagy in MGCs, we measured autophagy signaling during 48 h after FSH treatment *in vivo*. Immunohistochemical analysis indicated that FSH injection increased endogenous LC3 expression when compared with the control group (0 h; Figure 1a). In particular, LC3-positive staining was concentrated in the MGCs of antral and preovulatory follicles, both FSH-sensitive follicles. In addition, we labeled and tracked acidic organelles using lysotracker red staining. Results demonstrated that the fluorescence intensity was higher in follicles of FSH-treated mice (Figure 1b). Western blot results showed that the lipid conjugation of free LC3-I to the autophagic membrane-associated LC3-II was enhanced in MGCs following FSH treatment and that degradation of the autophagy receptor SQSTM1 (p62) was enhanced (Figures 1c and d). Thus, after intraperitoneal injection of FSH, autophagy signaling in MGCs from antral and preovulatory follicles was significantly increased and remained at a relatively high level.

### FSH promotes MGC autophagy through the AKT-mTOR pathway

**Figure 2:**
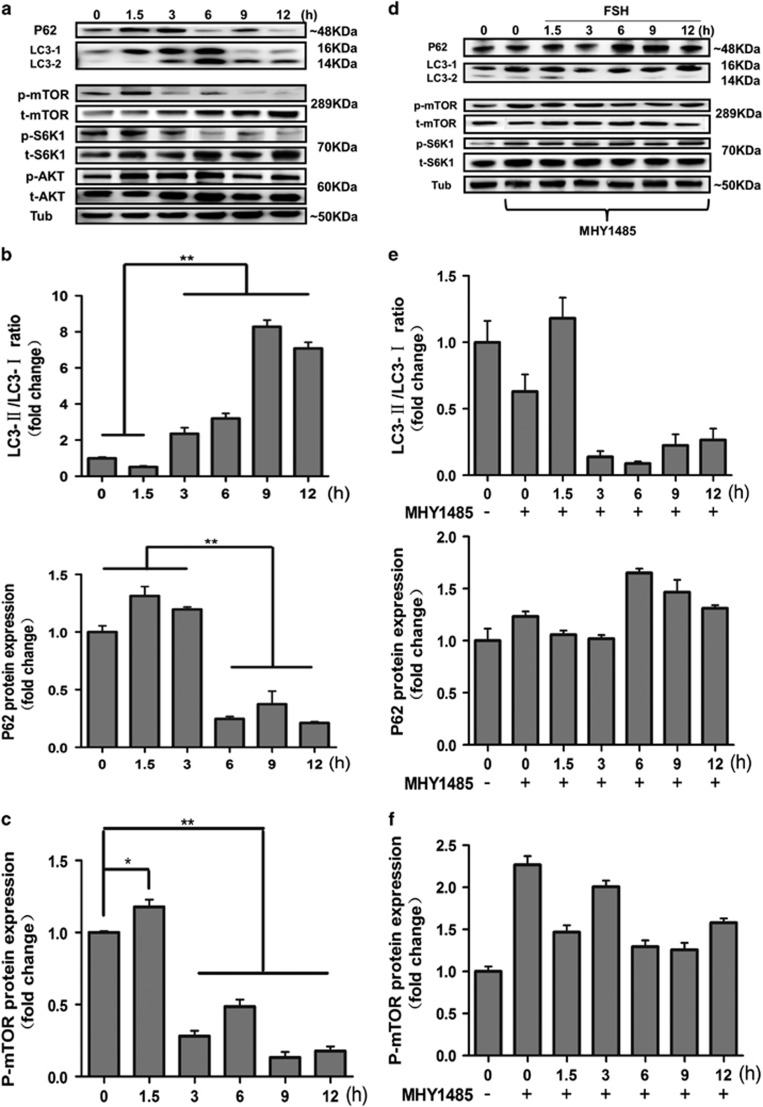
FSH regulates the AKT-mTOR pathway. (**a**) FSH increased the conversion of LC3-I into LC3-II and decreased the p62 protein level in MGCs at 12 h. The level of p-mTOR and p-S6K1 was increased at 1.5 h and decreased at 3, 6, 9, and 12 h compared to that in the control group. α-Tubulin was used as a loading control. (**b**) Quantitative analysis of protein level of LC3-II/LC3-I ratio and p62 in **a**, top. (**c**) Quantitative analysis of protein level of p-mTOR in **a**, bottom. (**d**) The effects of MHY1485 on MGCs autophagy induced by FSH injection at 12 h. The protein level of p-mTOR and p-S6K1 was increased after MHY1485 treatment. LC3-II/LC3-I ratio was decreased and the level of p62 was increased after MHY1485 treatment. α-Tubulin was used as a loading control. (**e**) Quantitative analysis of protein level of LC3-II/LC3-I ratio and p62 in **d**, top. (**f**) Quantitative analysis of protein level of p-mTOR in **d**, bottom. Data are presented as means±S.E of three experiments. *P<0.05. **P<0.01

To further investigate the molecular mechanism by which FSH induces autophagy in MGCs, we focused on molecular regulation within the 12 h following administration. As shown in Figures 2a and b, western blot results showed that LC3-II protein expression was significantly increased after FSH treatment and peaked at 6 h. Interestingly, the expression of LC3-I was significantly increased at 1.5 h (data not shown). We hypothesize that the activation of LC3-I is a preparation for cell autophagy because LC3-II can be distinguished from LC3-I by its increased mobility. The expression of p62 was increased within 3 h after administration and decreased over the next 9 h. These results demonstrated that autophagy is rapidly activated and maintained at a high level up to 12 h after administration. FSH activates multiple downstream signaling pathways in GCs, including PKA, PI3K, AKT-mTOR, p38-MAPK, and ERK1.^22^ Linking them, mTOR acts as a central sensor of growth factors, nutritional condition, and energy status, and plays a dominant role in autophagy.^23^ Our results indicated that AKT, mTOR, and S6K1 (downstream effector of mTOR) expression levels were enhanced by FSH stimulation when compared with the control group. The expression of p-AKT was induced at 1.5 h after FSH stimulation, but returned to the basal level at 9 h. p-mTOR and p-S6K1 expression levels were also induced at 1.5 h after FSH stimulation and then decreased significantly when compared to the control group (Figure 2a, bottom, Figure 2c). In addition, the effect of the mTOR activator, MHY1485, (10 mg/kg, 2 days) before FSH treatment was investigated. The results suggested that MHY1485 blocked the autophagy signaling induced by FSH. p-mTOR and p-S6K1 expression levels were maintained at a high level in the presence of MHY1485 (Figure 2d, bottom, Figure 2f), whereas LC3 expression showed no marked change compared to that in the control group (Figure 2d, top, Figure 2e). These findings demonstrated that FSH induces MGCs autophagy through the AKT-mTOR signaling pathway and initiates a dynamic process occurring within 12 h post-treatment.

### FSH upregulates HIF-1*α* and AMPK in MGCs

**Figure 3:**
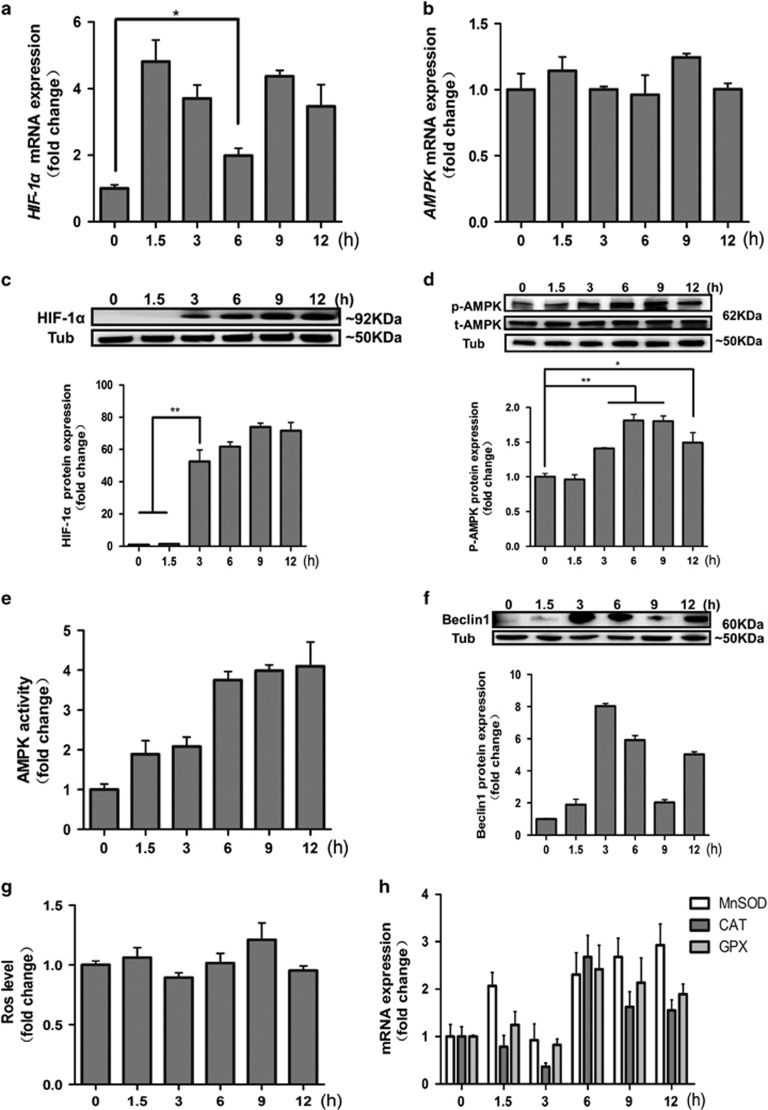
The effect of FSH on HIF-1*α* and AMPK in MGCs. (**a**) FSH injection increased *HIF-1α* mRNA level. The *HIF-1α* mRNA level was determined by real-time PCR. The relative expression data were normalized to the amount of *GAPDH*. (**b**) FSH treatment did not have an effect on AMPK mRNA expression. The *AMPK* mRNA level was determined by real-time PCR. *GAPDH* was used as an internal control. (**c**) FSH treatment increased HIF-1*α* protein expression at 3, 6, 9, and 12 h compared to 0 and 1.5 h. The relative expression data were normalized to *α*-Tubulin. (**d**) Western blot analysis of total AMPK and p-AMPK levels in MGCs after FSH injection at 12 h. *α*-Tubulin was used as a loading control. (**e**) AMPK activity was detected after FSH treatment. Detection was performed as described in Materials and Methods section. (**f**) Beclin1 protein expression in MGCs treated with FSH. Relative protein level was measured by densitometry and normalized to α-tubulin. (**g**) The cellular ROS level in MGCs after FSH treatment. Detection was performed as described in Materials and Methods section. (**h**) The effect of FSH on *MnSOD*, *CAT*, and *GPX* mRNA level determined by real-time PCR. The data are means±S.E; (*n*=3). *P<0.05. **P<0.01

FSH is a powerful growth factor that promotes GC proliferation,^24, 25^ as confirmed by our CCK-8 results during the 12 h period following FSH treatment (Supplementary Figure S1). Cell autophagy and apoptosis are tightly linked to cell metabolism. Excessive cell proliferation causes metabolic stress, including hypoxia and nutrition stress, promoting cell autophagy and death.^26^ Therefore, we investigated the expression of HIF-1a and AMPK by using qPCR and western blot. The results demonstrated that HIF-1*α* mRNA and protein expression was significantly upregulated (Figures 3a and c). Activation of AMPK was significantly enhanced between 3 and 12 h after FSH injection (Figures 3d and Figures 3e), while total AMPK expression did not change (Figures 3b and Figures 3d). In addition, the expression of a downstream factor, Beclin1, was also increased after FSH administration (Figure 3f). Recent reports indicated that reactive oxygen species (ROS) may lead to damage of cellular components and subsequently induce cell autophagy.^27, 28^ Therefore, we measured the intracellular ROS level in MGCs after FSH injection within 12 h. The level of intracellular ROS did not change significantly (Figure 3g). However, the mRNA levels of antioxidant enzymes, superoxide dismutase *(SOD)*, catalase *(CAT),* and glutathione peroxidase *(GPX),* increased (Figure 3h). These results demonstrated that FSH leads to hypoxia and reduces nutritional status in MGCs. Moreover, FSH plays a role in protecting GCs against the effect of ROS by activating the antioxidant enzyme system.

### HIF-1*α* is the critical factor in MGC autophagy

**Figure 4:**
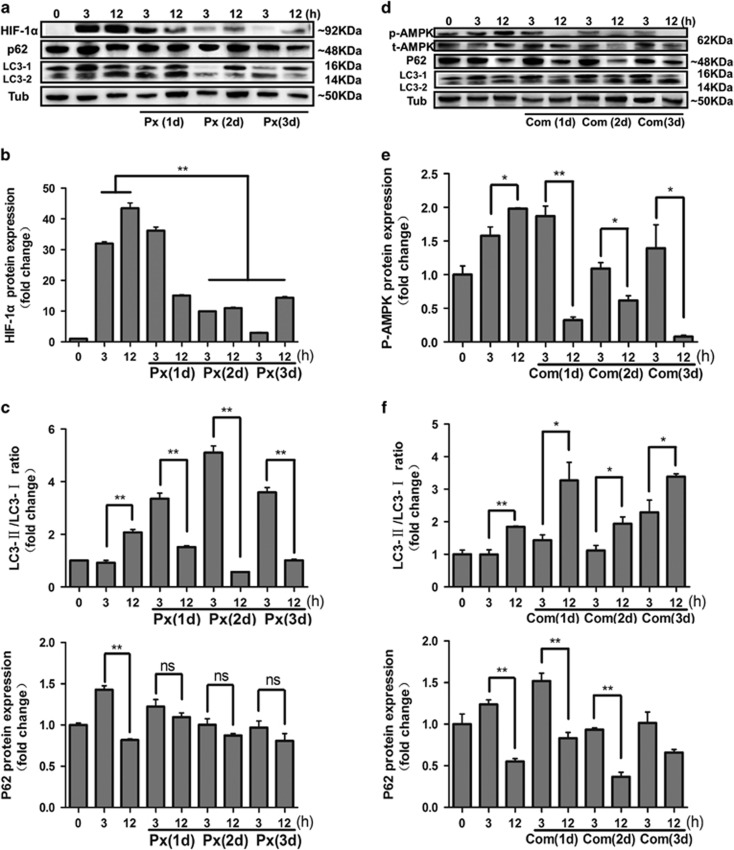
Blocking HIF-1*α* decreases FSH-induced autophagy in MGCs. (**a**) The effects of co-treatment of Px-478 with FSH on HIF-1*α*, p62, and LC3 protein levels, as detected by western blot. Relative protein levels were measured by densitometry and normalized to α-tubulin (**b**) Quantitative analysis of protein level of HIF-1*α* in **a**. (**c**) Quantitative analysis of protein level of LC3-II/LC3-I ratio and p62 in **a**. (**d**) The protein level of total AMPK, p-AMPK, p62, and LC3 after co-treatment of Compound C with FSH. Relative protein levels were normalized to α-tubulin. (**e**) Quantitative analysis of protein level of p-AMPK in **d**. (**f**) Quantitative analysis of protein level of LC3-II/LC3-I ratio and p62 in **d**. All experiments were performed in triplicate. *P<0.05. **P<0.01. Com, compound C; NS, not significant; Px, Px-478

To determine the effect of FSH-mediated HIF-1*α* and AMPK activation on cell autophagy, MGCs, with or without FSH, were treated with HIF-1*α* (Px-478) and AMPK inhibitors (Compound C), and cell autophagy signaling was detected by western blot. The experimental protocol is described in Supplementary Figure S2. After pretreating mice with Px-478, the expression of HIF-1*α* was significantly decreased at days 2 and 3 (Figures 4a and b). The LC3-II/LC3-I ratio was also significantly decreased at 12 h compared with that at 3 h after pretreatment with Px-478 (Figure 4c, top). In contrast, the expression of p62 was maintained at a high level after pretreatment with Px-478 (Figure 4c, bottom). Subsequently, we measured autophagy signaling in MGCs after AMPK inhibition. The results showed that the expression level of p-AMPK was inhibited by Compound C injection (Figures 4d and e) and total AMPK expression was inhibited. However, the LC3-II/LC3-I ratio and the degradation of p62 did not change compared with those in the groups only treated with FSH (Figure 4f), suggesting that the AMPK signaling pathway is not crucial for the promotion of cell autophagy although p-AMPK is highly expressed following FSH injection. These results demonstrated that HIF-1*α* is primarily involved in FSH-regulated MGC autophagy.

### FSH enhances MGC autophagy through HIF-1*α in vitro*

**Figure 5:**
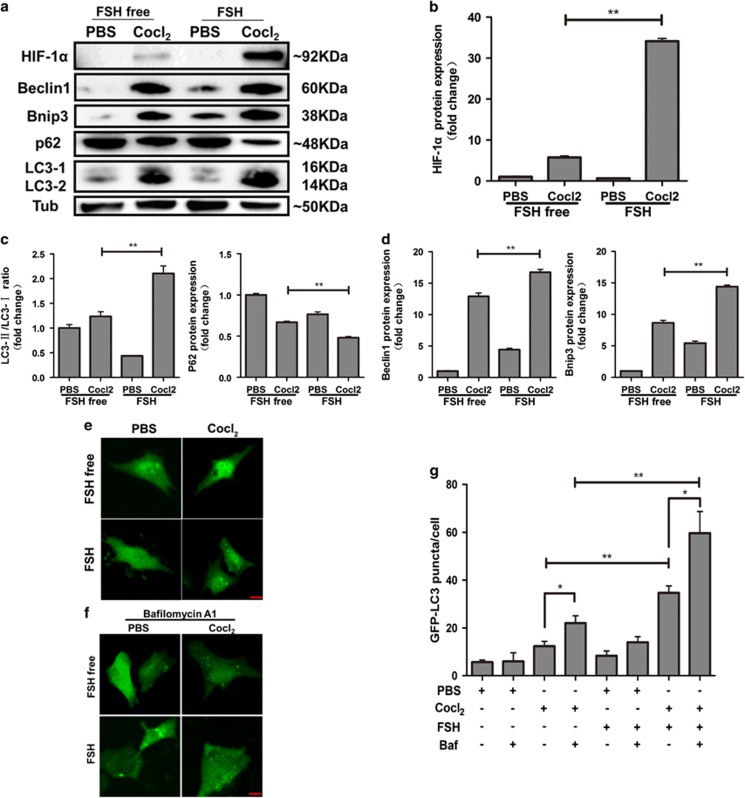
FSH promotes MGCs autophagy by regulating HIF-1*α in vitro*. (**a**) HIF-1*α*, Beclin1, Bnip3, p62, and LC3 protein levels in MGCs. After 4 h of cultured with CoCl_2_, the medium was replaced and FSH was added. Cells were harvested and the indicated proteins were detected by western blot after 6 h. Relative protein levels were normalized to α-tubulin. (**b**) Quantitative analysis of HIF-1*α* protein levels in **a**. (**c**) Quantitative analysis of LC3-II/LC3-I ratio and p62 protein levels in **a**. (**d**) Quantitative analysis of Beclin1 and Bnip3 expression in **a**. (**e**) MGCs were transfected with plasmid encoding GFP-LC3. Cells were treated with CoCl_2_ or FSH after 48 h and autophagy was assessed. Bar=10 *μ*m. (**f**) Autophagy flux in cells from (**e**). Cells were treated with Bafilomycin A1 (50 nM) for 4 h before analysis. Bar=10 *μ*m. (**g**) Quantitative analysis of the data in **e** and **f**. The data are mean±S.E.; (*n*=3). *P<0.05. ^**^P<0.01

To further confirm the effects of HIF-1*α* on MGC autophagy, we monitored this process in MGC primary cultures *in vitro*. Since HIF-1*α* is unstable under conditions of normoxia, we used a chemical inducer of HIF-1*α*, which acts by stabilizing HIF-1*α* transcription factor, inhibiting its degeneration under normoxia. As shown in Figures 5a and b, FSH in combination with CoCl_2_ significantly increased HIF-1*α* expression, suggesting that FSH functions as a positive regulator of HIF-1*α* expression. The ratio of LC3-II/LC3-I and p62 degradation increased in FSH-treated MGCs compared with that in the CoCl_2_-only group (Figure 5c). Consistent with the results presented in Figure 3f, in the presence of CoCl_2_, FSH significantly increased Beclin1 expression (Figure 5d, left). We also detected the expression of Bnip3, a member of the BH3-only protein family that contains a hypoxia response element in the promoter^29^ and is associated with autophagy in the hypoxia model.^30^ The results demonstrated that Bnip3 expression was significantly enhanced after treatment with FSH and CoCl_2_ compared to that in MGCs treated with CoCl_2_ alone (Figure 5d, right). Immunofluorescence studies indicated that FSH-induced HIF-1*α* significantly increased the formation of GFP-LC3 puncta, suggesting increased autophagy signaling under these conditions (Figures 5e and g). Moreover, we used the autophagy-flux inhibitor, Bafilomycin A1, which prevents lysosome degradation, thus increasing punctate GFP–LC3 exclusively when autophagy is active. Bafilomycin A1 treatment indicated that FSH significantly increased the autophagy flux, as monitored by GFP-LC3 puncta (Figures 5f and g). In addition, Bafilomycin A1 significantly increased the GFP-LC3 puncta in Cocl_2_ treated cells regardless of FSH. These results demonstrated that FSH promotes MGC hypoxia, further enhancing autophagy *in vitro*.

### Blocking HIF-1*α*, Beclin1, and Bnip3 attenuates FSH-induced autophagy in MGCs

**Figure 6:**
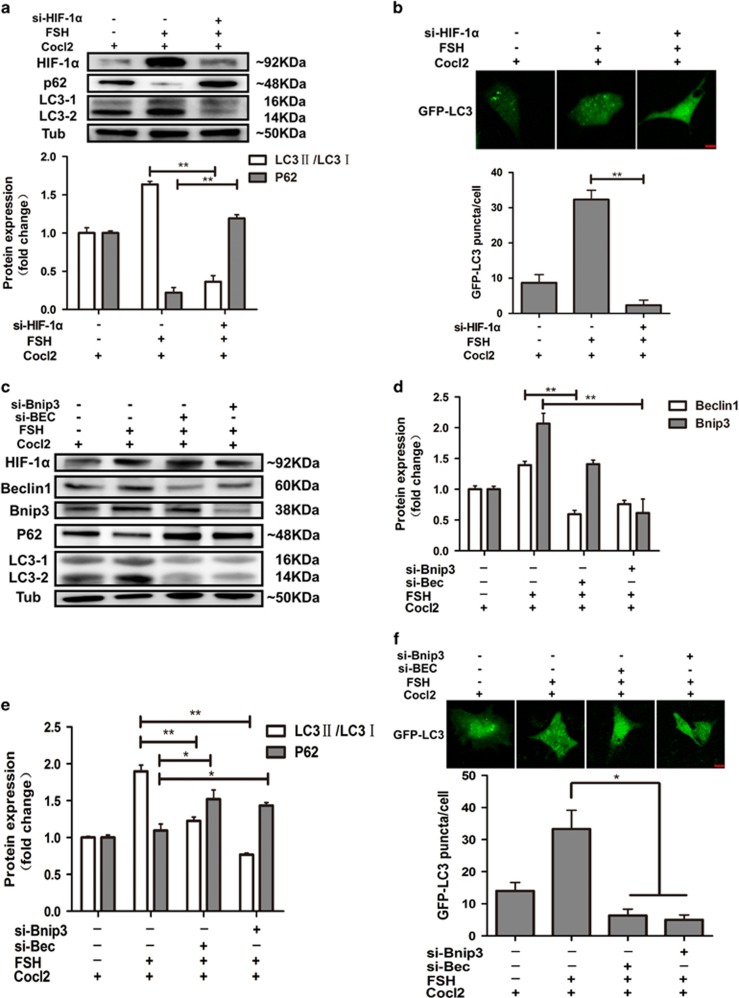
Knockdown of HIF-1*α*, Beclin1, and Bnip3 attenuates autophagy induced by FSH in MGCs. (**a**) HIF-1*α*, p62, and LC3 protein levels in MGCs. After MGCs were transfected with HIF-1*α* siRNA for 24 h, cells were treated with FSH or CoCl_2_. si-HIF-1*α*, siRNA-HIF-1*α*. Relative protein levels were normalized to α-tubulin. (**b**) MGCs were transfected with si-HIF-1*α* and GFP-LC3 plasmid, and GFP puncta were detected by immunofluorescence after FSH or CoCl_2_ treatment. Bar=10 *μ*m. (**c**) Western blot analysis of HIF-1*α*, Beclin1, Bnip3, p62, and LC3 protein levels in MGCs after transfection with siRNA. si-Bnip3, siRNA-Bnip3; si-Bec, siRNA-Beclin1. Relative protein levels were normalized to α-tubulin (**d**) Quantitative analysis of Bnip3 and Beclin1 protein levels in **c**. (**e**) Quantitative analysis of LC3-II/LC3-I ratio and p62 protein levels in **c**. (**f**) MGCs were transfected with si-Bnip3 or si-Beclin1 together with GFP-LC3 plasmid. Cell autophagy was detected after 48 h with FSH or CoCl_2_ treatment. Bar=10 *μ*m. All experiments were performed in triplicate. *P<0.05. ^**^P<0.01

To further test whether loss of HIF-1*α* function decreases autophagy in MGCs after co-treatment with FSH and CoCl_2_, si-HIF-1*α* (siRNA HIF-1*α*) was used to knockdown HIF-1*α* expression induced by FSH. MGCs were transfected with si-HIF-1*α* and then treated with FSH and CoCl_2_. HIF-1*α* expression was inhibited by si-HIF-1*α* (data not shown). In addition, autophagy signaling was decreased (Figure 6a). Consistently, transfection with si-HIF-1*α* also decreased GFP-LC3 puncta observed by immunofluorescence (Figure 6b). Next, we investigated the role of downstream factors of HIF-1*α* (Beclin1 and Bnip3) on autophagy. Two siRNA (si-Bec and si-Bnip3) were used to knockdown Beclin1 and Bnip3 expression. Results demonstrated that Beclin1 and Bnip3 expression was significantly suppressed after transfection with siRNA (Figures 6c and d). Autophagy signaling was inhibited, even under conditions of hypoxia as indicated by decreased LC3-II/LC3-I ratio, inhibited p62 degradation, and decreased formation of GFP-LC3 puncta (Figures 6e and f). In addition, the differences between FSH treated and untreated HIF1*α*, Beclin1, and Bnip3 down-regulated cells are not significant (Supplementary Fig. S3). Together, these results indicated that Beclin1 and Bnip3 are critical factors in FSH-induced autophagy, mediated by modulation of HIF-1*α*.

### FSH has a protective effect on follicular development by increasing the activity of mitochondrial clearance

**Figure 7:**
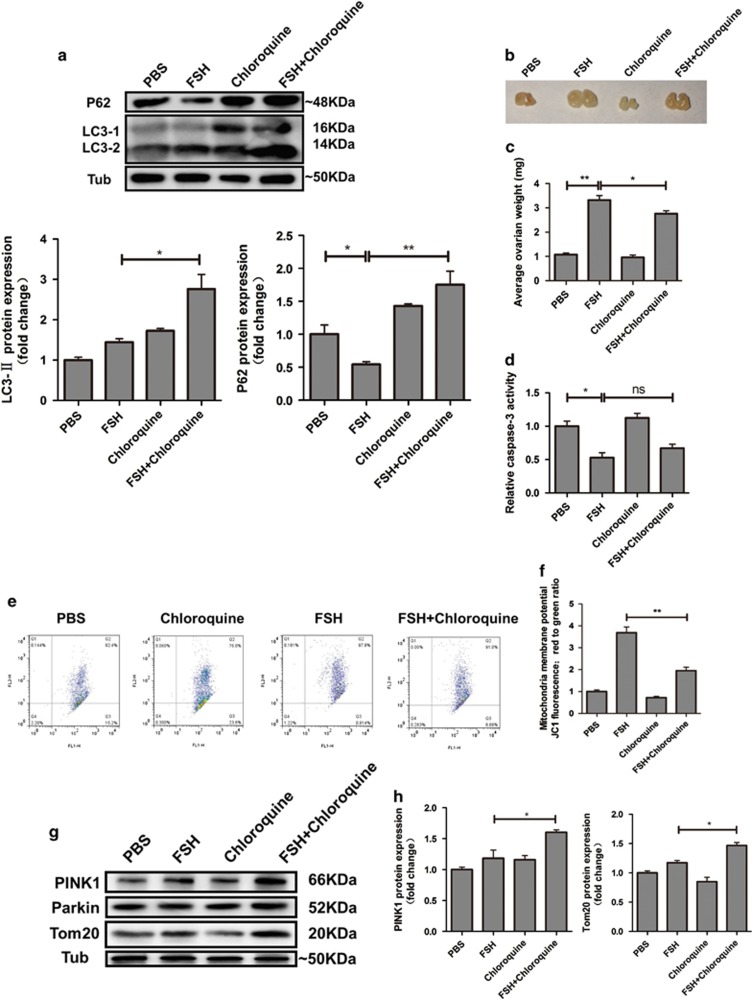
Blocking autophagy affects mitochondrial membrane potential. (**a**) Mice treated with or without chloroquine for 5 days were then treated with FSH for 12 h, the expression of LC3 and p62 in MGCs was determined by western blotting. (**b**) Ovaries derived from mice treat with or without chloroquine and FSH. (**c**) The effect of autophagy on follicle was quantified by calculating the average ovarian weight. (**d**) The relative caspase-3 activity after chloroquine and FSH treatment. Detection was performed as described in Materials and Methods section. (**e**) Mitochondrial membrane potential was measured by JC-1 staining and analyzed by flow cytometry. The upper right fraction was labeled by JC-1 as JC-1 red (intact fraction) and the lower right fraction was labeled by JC-1 as JC-1 green (damaged fraction), respectively. (**f**) Quantitative analysis of the data in **e**. (**g**) The protein expression of PINK1, Parkin, and Tom20 was determined by western blotting. Relative protein levels were normalized to α-tubulin. (**h**) Quantitative analysis of the data in **g**. The data are means±S.E; (*n*=3). *P<0.05. ^**^P<0.01. NS, not significant

We next investigated the relationship between FSH-induced autophagy and follicular development. Chloroquine, an autophagy inhibitor, induced the accumulation of LC3-II and p62 in FSH-treated GCs by blocking the later stage of autophagy (Figure 7a). Interestingly, FSH treatment significantly increased the size and weight of the ovary, which were reduced by chloroquine (Figures 7b and c). However, MGC apoptosis was not significantly affected by autophagy inhibition, assessed by monitoring caspase-3 activity, and the expression of apoptosis-related genes (Figure 7d and Supplementary Figure S4). Previous reports indicated a mutual regulation between apoptosis and autophagy in the death signaling process mediated by mitochondria.^31, 32^ Thus, we investigated whether FSH-mediated autophagy affected mitochondrial membrane potential (ΔΨ (m)). JC-1 staining experiments indicated that the increased mitochondrial membrane potential by FSH was abolished by autophagy inhibition (Figures 7e and f). In addition, we assessed the expression of the PINK1-Parkin system that belongs to the mitophagy pathway. Western blot indicated that PINK1 expression increased in MGC treated with FSH, and PINK1 level further increased after chloroquine treatment (Figures 7g and h). However, no marked change of Parkin expression was detected (Figure 7g), as Parkin could promote mitophagy by changing the mitochondrial location.^33^ Moreover, the loss of mitochondria resulting from mitophagy was decreased, which was indicated by mitochondrial membrane protein, Tom20 protein expression (Figures 7g and h). Taken together, these data demonstrated that mitophagy induction via the PINK1-Parkin pathway is an important mechanism of FSH-mediated follicular growth and development.

### FSH-induced autophagy in MGCs is associated with cell proliferation

**Figure 8:**
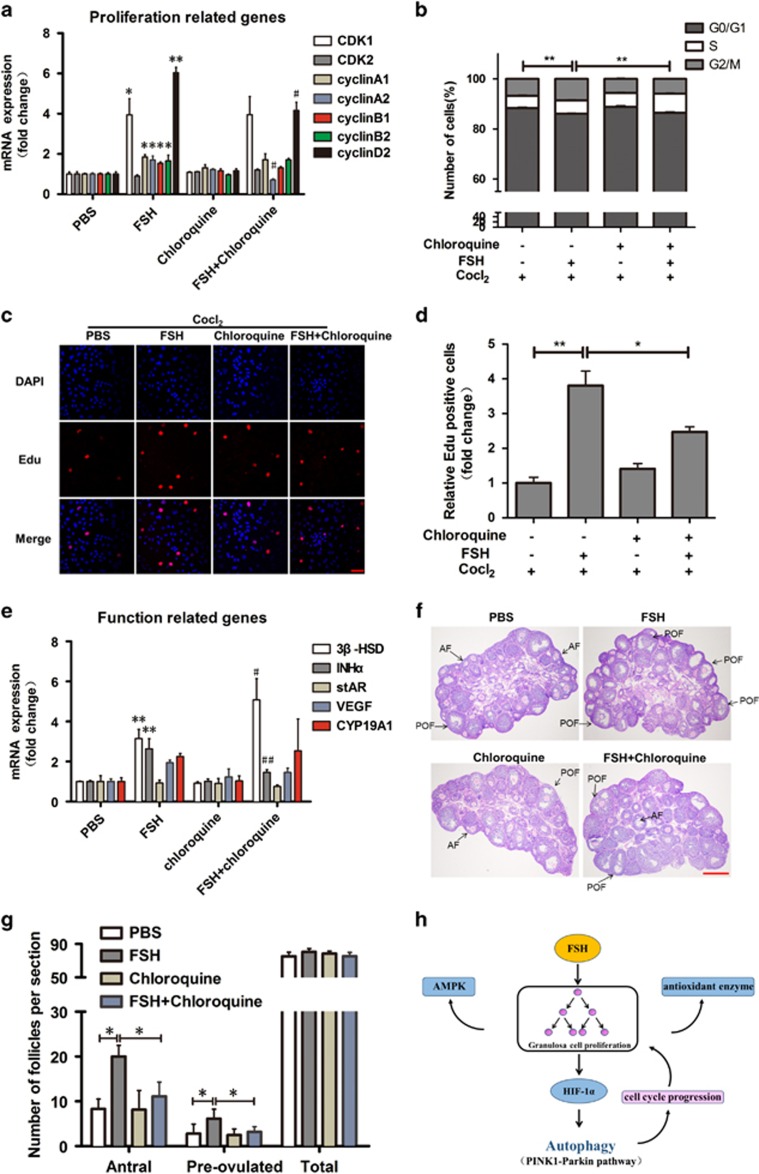
FSH-induced autophagy promotes MGC proliferation. (**a**) Mice treated with or without chloroquine for 5 days were then treated with FSH for 12 h, the proliferation related genes were determined by qPCR. The relative expression data were normalized to the amount of *GAPDH*. Significances were marked as *P<0.05. ^**^P<0.01 *versus* PBS group; ^#^*P*<0.05 *versus* FSH group. (**b**) Analysis of cell proliferation. Cells treated with Cocl_2_ and FSH were incubated with chloroquine at 24 h before the detection. The cell cycle profile was analyzed by flow cytometry using Propidium Iodide (PI). (**c**) MGCs treated as described in **b** were labeled with EdU, EdU-positive cells, red; cell nuclei, blue; Bar=50 *μ*m. (**d**) Quantitative analysis of the data in **c**. (**e**) The function related genes were determined by qPCR. *GAPDH* was used as an internal control. Significances were marked as ^**^P<0.01 *versus* PBS group; ^#^*P*<0.05. ^##^*P*<0.01 *versus* FSH group. (**f**) Representative H&E staining of ovaries. Bar=500 *μ*m. *AF*, antral follicle; *POF*, pre-ovulated follicle. (**g**) Quantitative analysis of the data in (**f**). The data are means±S.E; (*n*=3). *P<0.05. ^**^P<0.01. (**h**) Schematic representation of FSH regulation of autophagy in MGC. FSH-mediated cell proliferation promotes HIF-1*α* expression, which further leads to autophagy in hypoxic condition. Autophagy transfers the hypoxic stress through the mitophagy pathway and participates in the regulation of the cell cycle, which has a positive feedback on cell proliferation

Autophagy is required for the cyclic phase of GC proliferation and differentiation.^34^ Thus, we investigated the potent function of autophagy in FSH-mediated cell proliferation. qPCR results revealed that cell proliferation was inhibited, mainly reflected in *cyclinA2* and *cyclinD2* expression (Figure 8a). To better assess cell proliferation deregulation, cell cycle, and cell proliferation were investigated. Flow cytometry showed that FSH and chloroquine co-treatment resulted in a small population of cells in the G2/M phase when compared with the FSH-treated group (Figure 8b), suggesting a cell cycle delay in S phase caused by autophagy inhibition. As shown in Figure 8c, chloroquine significantly attenuated the increased number of EdU-labeled cells following FSH treatment (Figures 8c and d), as well as the number of living/viable cells detected by CCK-8 assay (Supplementary Figure S5). Therefore, FSH-induced autophagy promotes MGC proliferation and follicular development. In addition, the expression of genes involved in estrogen biosynthesis and steroidogenic regulation associated with follicle development was detected by qPCR. Interestingly, the expression of *3β-HSD* was enhanced. In contrast, *INHα* expression was decreased compared with FSH only treatment (Figure 8e). To further demonstrate that autophagy is necessary for the development of follicles, we performed a hematoxylin and eosin (H&E) staining assay. The increased number of antral follicles and preovulatory follicles after FSH treatment was reduced by chloroquine (Figures 8f and g). Overall, these results provided evidence that FSH-mediated autophagy is associated with GC proliferation and follicular development.

## Discussion

During follicular development, a proportion of ovarian follicles are removed by atresia prior to maturation in order to promote energy investment and ovulation of viable follicles. Autophagy captures and degrades intracellular components such as abnormal proteins and damaged organelles to sustain metabolism and homeostasis. In particular, autophagy is closely related with the remodeling of follicle cells during the process of follicular development.^35, 36^ Signs of autophagy depend on gonadotropin dose, age, and body weight in freshly harvested human GCs,^37^ suggesting that autophagy is common in mammalian ovaries where most follicles and cells are in a highly regulated state, balancing hormonal and environmental stimulus. However, the underlying molecular mechanism is still unknown.

FSH is an important survival factor leading to selection and survival of growing follicles during development.^38^ The physiological functions of FSH are achieved by activating several signaling cascades in GCs, including PKA, PKB, p38-MAPK, and ERK1/2, which in turn modulate >100 different target genes.^39^ These downstream factors are directly or indirectly involved in autophagy regulation.^40, 41, 42, 43^ In the mouse ovary, TGF*β* signaling was activated after exposure to FSH and LH.^44^ Inhibition of TGF*β* signaling by blocking TGF*β*R2 significantly decreased FSH-mediated autophagy signaling.^45^ In porcine GCs, FSH increased the level of inhibitor of NF-*κ*B (I*κ*B) protein and subsequently increased autophagy via JNK signaling.^46^ In this study, we demonstrated that FSH administration enhanced autophagy in MGCs. Injection of FSH increased endogenous LC3 staining, the LC3-II/LC3-I ratio, p62 degradation, and enhanced lysotracker staining in MGC. These are the most widely used tests for autophagy determination.^47^

mTOR is a downstream regulator of PKB, which senses nutrient, energy and oxygen availability, and growth factor signaling, and plays an important role in autophagy.^48^ In this study, we introduced mTOR as the main factor affecting autophagy induced by FSH in MGCs. FSH activates the expression of p-mTOR and promotes the accumulation of LC3-I within 1.5 h. After 3 h, FSH significantly enhanced signs of autophagy by inhibiting the expression of p-mTOR. In addition, the mTOR activator, MHY1485, suppressed the autophagy level following FSH treatment. These results suggest that FSH regulates autophagy through mTOR inactivation.

Hypoxia is a pathological condition in which the body or a region of the body is deprived of an adequate oxygen supply. Evidence suggests that HIF-1*α* plays an essential role in angiogenesis,^49^ cell proliferation/survival,^50^ and glucose/iron metabolism.^51, 52^ Consistent with our results (Figure 2a,Figures 3a and c), a previous study indicated HIF-1*α* is activated in FSH-stimulated ovarian cancer cells, SKOV-3, and that the PI3K/AKT signaling is upregulated.^53^ In the ovary, excessive cell proliferation induced by gonadotropins promotes the accumulation of HIF-1*α* and leads to hypoxia.^54^ Here, we identified HIF-1*α* as an inducible factor after FSH treatment. Injection of FSH significantly increased HIF-1*α* expression *in vivo* and *vitro*. Our results are consistent with a recent report demonstrating that HIF-1*α* is a downstream factor of FSH in rat GCs.^55^ Remarkably, *HIF-1α* mRNA decreased at 6 h without reflecting a modulation of HIF1-*α* protein level until 12 h (Figures 3a and c), indicating that HIF-1*α* transcriptional and post-transcriptional regulation is also involved in this process.

Recent studies indicated that autophagy has a complex and close link with hypoxia.^56, 57^ Evidence from various mammalian cell types indicates that HIF-1*α* induces autophagy by activating Bnip3.^58, 59^ The expression of this conserved member of the BH3 only subfamily of the pro-apoptotic Bcl-2 family proteins correlates with the induction of autophagy in different cell models.^60, 61, 62, 63^ Moreover, Bnip3 can dissociate Beclin1 from the Bcl-2–Beclin1 complex,^64^ consistent with our results showing that Bnip3 knockdown affects Beclin1 expression (Figure 6d). Beclin1 is an important component of the class III PtdIns3K complex, required for the induction of autophagy. Hypoxia preconditioning in hepatocellular carcinoma significant activated autophagy, but this process can be attenuated by Beclin1 knockdown.^65^ In this study, Bnip3 and Beclin1 expression was significantly enhanced in MGCs subjected to hypoxia. Furthermore, Bnip3 or Beclin1 knockdown decreased autophagy signaling in MGCs, suggesting that Bnip3 and Beclin1 regulation is involved in hypoxia-induced autophagy.

During follicular development, the constituent cells, including theca cells, GCs, and oocytes, exhibit a series of functional gene activation. A cohort of follicles that have undergone initial development are stimulated to develop further by rising concentrations of gonadotropins. The GCs become more responsive to FSH and show a higher rate of proliferation once a follicle is selected as a dominant follicle. Therefore, autophagy appears to be an important, evolutionarily conserved mechanism for maintaining homeostasis and providing energy.^66^ A previous report showed that autophagy was upregulated during cyclic phases of cell proliferation and differentiation.^34^ In addition, Atg3-, Atg5-, or Pik3c3/Vps34-deficient T cells cannot efficiently proliferate because of loss of the cell cycle inhibitor, CDKN1B/p27Kip1, which is selectively degraded by autophagy.^67^ In this study, inhibition of autophagy reduced the proliferation rate of MGCs, further decreasing the number of antral follicles and preovulatory follicles.

Once the dominant follicle is mature, GCs begin to express aromatase, to secrete estradiol and increasing the amount of inhibin B. Thus, maintaining the homeostasis of the ovary to produce more mature follicles and prevent follicles from entering atresia is important. Previous results highlighted a critical role for autophagy in the function of GCs. Autophagy inhibition by conditional knockout of Beclin1 in the ovarian GC population causes a defect in progesterone production and a subsequent preterm labor phenotype.^68^ In porcine GC, autophagy activated through NF-*κ*B inhibition promotes steroidogenesis.^46^ In this study, there was a change in the transcription of progesterone biosynthesis genes, 3 beta-hydroxysteroid dehydrogenase (3*β*-HSD) and inhibin alpha subunit (INH*α*), representing a crosslink with the molecular regulation of autophagy. Although no change was observed in the levels of CYP19A1, controlling estradiol production, autophagy may still be involved in estradiol signaling, as demonstrated in different cell lines.^69, 70, 71^

Reports revealed that autophagy is associated with GC apoptosis and follicle atresia throughout the reproductive period.^13, 18^ In this study, although no significant change was observed in apoptosis-related gene expression after autophagy inhibition, the mitochondrial membrane potential was significantly changed through the PINK1-Parkin system, indicating that a selectively autophagy, mitophagy was involved in this process. Since mitophagy facilitates cell death programs,^72^ we speculate that FSH-induced mitophagy has a protective role in damaged mitochondrial clearance. Interestingly, a recent report indicated that defect of mitochondrial fusion protein Mfn2 impaired autophagy-induced degradation, subsequently decreasing mitochondrial oxygen consumption rate and cell glycolysis, reducing ATP production, and suppressing cell proliferation.^73^ Therefore, FSH-induced autophagy is a regulatory mechanism that ensure the order and timing of cell cycle transition by mitophagy activation through the PINK1-Parkin pathway (Figure 8h), which may help reducing follicle atresia and GC apoptosis.^74^

In summary, FSH treatment promotes the activation of autophagy via upregulation of HIF-1*α* in MGCs. FSH-mediated autophagy has a protective role on GC proliferation and follicle development through the selective degradation of damaged mitochondria. Blocking FSH-induced autophagy influences steroid production and antral/preovulatory follicle numbers. Overall, our study highlights a mechanism by which FSH regulates MGC autophagy, which may be a novel strategy to reduce follicle atresia and degeneration.

## Materials and methods

### Animal treatment

All animal experiments were approved by Nanjing Agricultural University, Animal Research Institution Committee. Three to 4-week-old female ICR mice (Nanjing Qinglongshan Experimental Animal Center) were housed, five per cage, in a temperature controlled (22±2 °C) room with a 12: 12 h light: dark cycle (lights on from 07 00 to 1900  hours) and free access to water and food. To induce MGC autophagy, mice were injected i.p. with FSH (Ningbo Second Hormone Factory, Ningbo, China) on four successive occasions (10, 10, 5, and 5 IU) at 12 h intervals. MGCs were isolated from dominant follicles (DFs; >200 *μ*m) in the left ovaries of each mouse, for qRT-PCR and immunoblotting. The right ovaries were fixed with 4% paraformaldehyde and embedded in paraffin for subsequent immunohistology and lysotracker staining. For activator and inhibitor experiments, MHY1485 (10 mg/kg, 2 days) and chloroquine (20 mg/kg, 5 days) obtained from Sigma (St. Louis, MO, USA) were injected before FSH administration. HIF-1*α* inhibitor, Px-478, and AMPK inhibitor, compound C, (Selleck Chemicals, Houston, TX, USA) were injected before FSH treatment and the experiment protocol is described in Supplementary Figure S2.

### Immunohistology

Mouse ovaries used for histological analysis were fixed with 4% paraformaldehyde overnight at 4 °C, dehydrated, and embedded in paraffin. Ovarian sections (5-*μ*m thickness) were incubated with anti-LC3 rabbit antibody (1:300; #L8918, Sigma-Aldrich), followed by incubation with a biotinylated secondary antibody (#B7151, Sigma-Aldrich) for 1 h at a dilution of 1:500. For lysotracker staining, ovarian sections pretreated as above were incubated for 2 min with 100 *μ*M Lysotracker Red (Beyotime Institute of Biotechnology, Haimen, China) in phosphate-buffered saline (PBS). For H&E staining, the slides were stained with H&E after deparaffinization. The sections were dehydrated, mounted, and examined using a dotSlide digital virtual microscopy system (Olympus, Tokyo, Japan).

### Western blot and antibodies

Cells were harvested by using radioimmune precipitation assay lysis buffer (Pierce Chemical, Rockford, IL, USA) and protein was quantified by the BCA method (Pierce, Chemical). Cell lysates containing 25 *μ*g total protein were fractionated by using SDS-PAGE and transferred onto PVDF membranes (Millipore, Billerica, MA, USA). After blocking with 5% BSA in Tris-buffered saline containing Tween (TBST) for 1 h, membranes were incubated with primary antibody in TBST overnight at 4 °C. The antibodies, LC3 (1:1000; #L8918) was from Sigma-Aldrich, p62 (1:1000; #5114), AMPK (1:1000; #5832), AMPK (phosphor-Thr172) (1:1000; #2535), Bnip3 (1:1000; #3769), p70S6K (1:1000; #2708), p70S6K (phosphor-Thr389) (1:1000; #9206), and Parkin (1:1000; #2132) were purchased from Cell Signaling Technology (Danvers, MA, USA). Anti-AKT (1:1000; #ab18785), anti-AKT (phospho-ser473) (1:1000; #ab66138), anti-mTOR (1:1000; #ab2732), anti-mTOR (phospho-ser2448) (1:1000; #ab1093), anti-HIF-1*α* (1:1000; #ab179483), anti-PINK (1:1000; #ab23707) were obtained from Abcam (Cambridge, UK). Anti-Beclin1 (1:500; #sc-11427) and anti-Tom20 (1:500; #sc-11021) were from Santa Cruz Biotechnology (Santa Cruz, CA, USA). Subsequently, the membrane was incubated in HRP-conjugated anti-rabbit secondary antibody (1:2000; #7074, Cell Signaling Technology) or anti-mouse secondary antibody (1:2000; #7076, Cell Signaling Technology) for 2 h at room temperature. After washing, the membrane was processed by using SuperSignal West Pico chemiluminescent substrate (Pierce Chemical). As an internal control, *α*-tubulin was detected by using an anti-tubulin antibody (1:2000; #T5168, Sigma-Aldrich).

### Quantitative RT-PCR (qRT-PCR)

Total RNA was extracted by using TRIZOL (Invitrogen, Carlsbad, CA, USA) and reverse-transcribed into cDNA using Moloney murine leukemia virus RT according to the manufacturer’s protocol (Bio-Rad, Hercules, CA, USA). Real-time PCR was performed with SYBR Premix Ex Taq (Takara Bio, Tokyo, Japan) in a reaction volume of 20 *μ*l and the ABI StepOne system (Applied Biosystems, Foster City, CA, USA). Primer sequences are listed in Appendix: Supplementary Table S1. Melting curves were analyzed to verify amplification specificity. Expression data were normalized to the amount of *GAPDH* expressed.

### Cell proliferation assay

The proliferation of MGCs was determined by using a Cell Counting Kit-8 (CCK-8) (Dojindo Laboratories, Japan) and a 5-ethynyl-2′-deoxyuridine (EdU) assay using an EdU assay kit (Ribobio, Guangzhou, China) according to the manufacturer’s protocol. In brief, cells were plated into 96-well plates at a concentration of 5 × 10^3^ cells/well. After treatment as indicated, cells were collected and seeded into a 96-well plate. CCK-8 solution (10 *μ*l) was added to each well, followed by incubation for 2 h at 37 °C. The absorbance at 450 nm was determined by using a multiplate reader (Lambda Bio-20; Beckman, La Brea, CA, USA). The cell viability was calculated by the optical density (OD) values of treated groups/OD values of control group × 100%. For the EdU assay, 25 *μ*M EdU was added to the cells, and the cells were incubated for 2 h at 37 °C. The cells were then fixed with 4% paraformaldehyde for 15 min at room temperature and exposed to 0.5% Triton X-100 for 20 min. After 3 washes with PBS, the cells were stained with 100 *μ*l of Apollo Dye Solution for 30 min. The nucleic acids in all of the cells were stained with DAPI. Images were taken by using a fluorescence microscope (Carl Zeiss, Germany). All experiments were performed in triplicate.

### Intracellular ROS measurement

Following FSH treatment, follicular GCs were collected by puncture of the dominant ovarian follicle (>200 mm) in the ovary. Levels of ROS in cells were measured by using the GENMED cellular superoxide anion colorimetric quantitative determination kit (GENMED, Shanghai, China). All procedures were performed according to the manufacturer’s instructions.

### AMPK activity assay

After FSH treatment, cells were harvested and the AMPK activity was measured at an absorbance of 595 nm according to the manufacturer’s protocol (GENMED, Shanghai, China). The AMPK experiments were conducted in triplicate, and the results were normalized to cell protein concentration.

### MGC culture

Mice were injected intraperitoneally with 10 units of PMSG^75^ and euthanized 44 h later. Superovulated mouse ovaries were obtained and transferred to petri dishes (35 × 15 mm) filled with PBS, then punctured with a syringe to release MGCs from DFs (>200 *μ*m in diameter) under a surgical dissecting microscope. MGCs (1 × 10^6^) were plated into T25 flasks in 4 ml of Dulbecco’s Modified Eagle’s Medium: Nutrient Mixture F-12 (1:1; Life Technologies, Carlsbad, CA, USA) supplemented with 15% fetal bovine serum (Life Technologies) and 1% antibiotics (100 IU/ml penicillin and 100 *μ*g/ml streptomycin; Life Technologies). To induce cell hypoxia, 200 *μ*M CoCl_2_ (Sigma-Aldrich) was added to the culture medium at a concentration of 150 *μ*M. Induced cells were harvested for different assays.

### Cell transfection

HIF-1*α* siRNA, Beclin1 siRNA, and Bnip3 siRNA were purchased from Santa Cruz Biotechnology (#sc-35562; #sc-29798; and #sc-37452). GFP-LC3 plasmid was kindly provided by Jiyong Zhou of Zhejiang University, Zhejiang, China. Transfections were performed by using Lipofectamine 2000 (Invitrogen) following the manufacturer’s instructions. The medium was replaced 5 h after transfection.

### GFP-LC3 assay

MGCs were seeded into 24-well plates post-treatment, the coverslips were washed, mounted on slides, and inspected under a confocal laser scanning microscope (Carl Zeiss, Göttingen, Germany). Several bright green fluorescent puncta were observed in the cells. One punctum was considered equal to one autophagosome. The results are presented as the average number of puncta per cell.

### Caspase-3 activity assay

The activity of caspase-3 was determined by using the Caspase-3 activity kit (Beyotime Institute of Biotechnology) according to the manufacturer’s instructions. After treatment, GCs were homogenized in 100 ml reaction buffer (1% NP-40, 20 mM Tris–HCl (pH 7.5), 137 mM Nad, and 10% glycerol) containing 10 mL caspase-3 substrate (Ac-DEV D-pNA) (2 mM). Lysates were incubated at 37 °C for 2 h. Samples were measured with a microplate reader at an absorbance of 405 nm.

### JC-1 assay

The lipophilic cation JC-1 was used to assess the mitochondrial status of MGC. According to the manufacturer (Beyotime Institute of Biotechnology), JC-1 reversibly changes its fluorescence from green (monomeric status) to orange (multimeric status) when the mitochondrial membrane potential is high.

After treatment, 1 × 10^6^ cells of each group were collected and incubated with 10 *μ*g/ml of 5, 5′, 6, 6′-tetrachloro-1, 1′, 3, 3′-tetraethylimidacarbocyanine iodide (JC-1) at 37 °C, 5% CO2 for 30 min. The cells were analyzed by using a BD FACScan system (Becton Dickinson, Franklin, NJ, USA).

### Cell cycle assay

Cells were harvested, centrifuged at 12000 r.p.m. for 5 min, and washed three times with cold PBS. Subsequently, the cells were fixed in 70% ice cold ethanol overnight at 4 °C. After 30 min digestion with 1 *μ*g/*μ*l RNase, the cells were resuspended in 250 *μ*l of propidium iodide staining solution (10 *μ*g/ml) and incubated for 1 h at room temperature in the dark. The distribution of cells in the G0/G1, S, and G2/M phase was determined following analysis on a BD FACScan system (Becton Dickinson).

### Follicle classification and counting

Ovaries were cut into 3–5 mm-thick sections, deparaffinized, hydrated through ethanol series, and stained with H&E. Ovarian follicles at different developmental stages were counted in collected sections of an ovary, based on the well-accepted standards established by Pedersen and Peters.^76^ In brief, follicles were classified as antral follicles when antrum formation was visible. Follicles presenting a rim of cumulus cells surrounding the oocyte were classified as preovulatory follicles.

### Statistical analysis

Data were analyzed by using SPSS version 18.0 (SPSS Inc., Chicago, IL, USA). Statistical significance was calculated with the Student’s *t*-test (*P*<0.05 was considered statistically significant). All experiments were repeated at least three times. Results are expressed as the mean±S.E.

## Publisher’s Note

Springer Nature remains neutral with regard to jurisdictional claims in published maps and institutional affiliations.
