# Supplementary figures and images for: Administration of follicle-stimulating hormone induces autophagy via upregulation of HIF-1α in mouse granulosa cells
Source: Cell Death Dis. 2017 Aug 17;8(8):e3001–. doi: 10.1038/cddis.2017.371 (PMC5596559; doi:10.1038/cddis.2017.371)

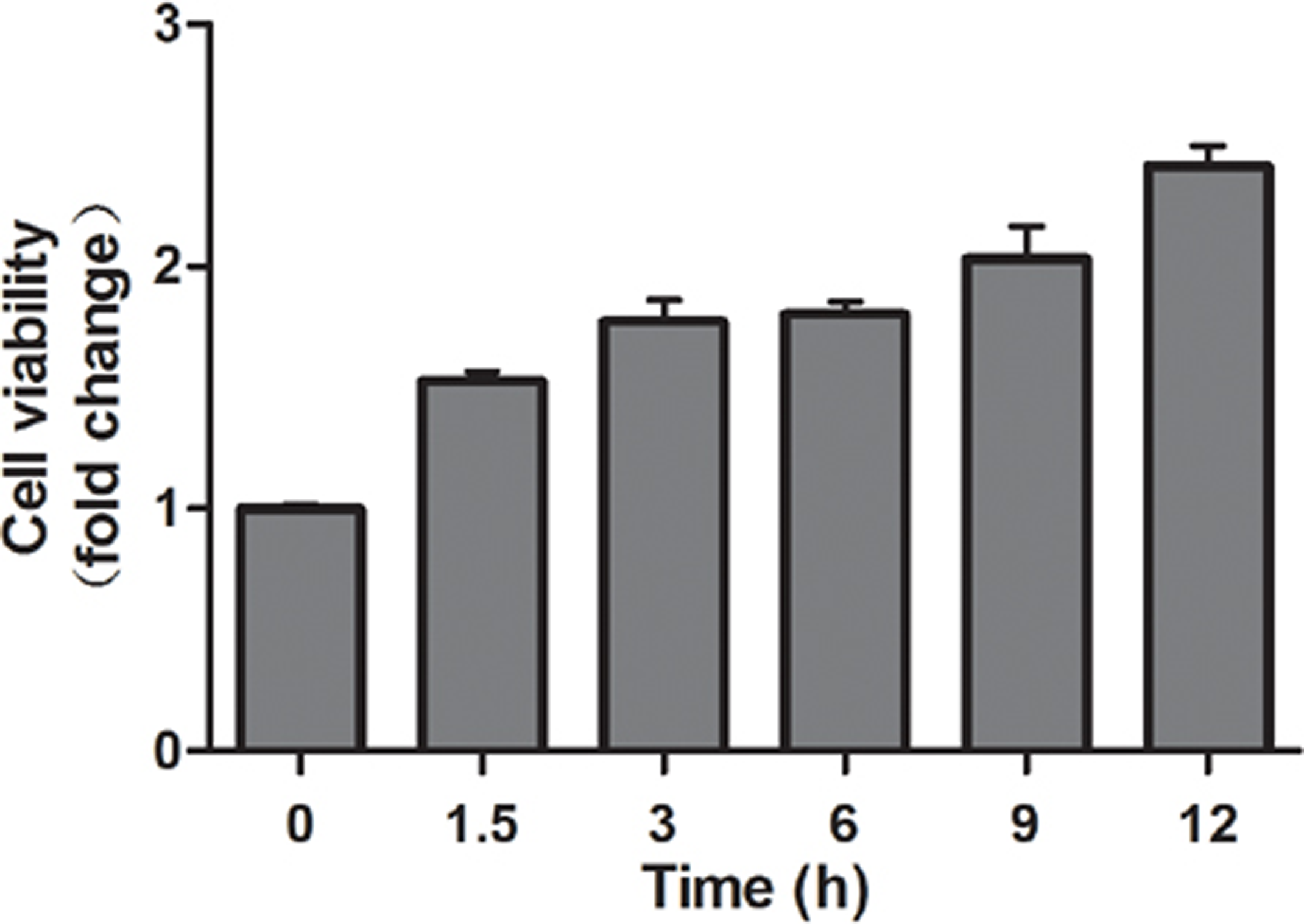

Supplement: Supplementary Figure S1 [file cddis2017371x1.tif]

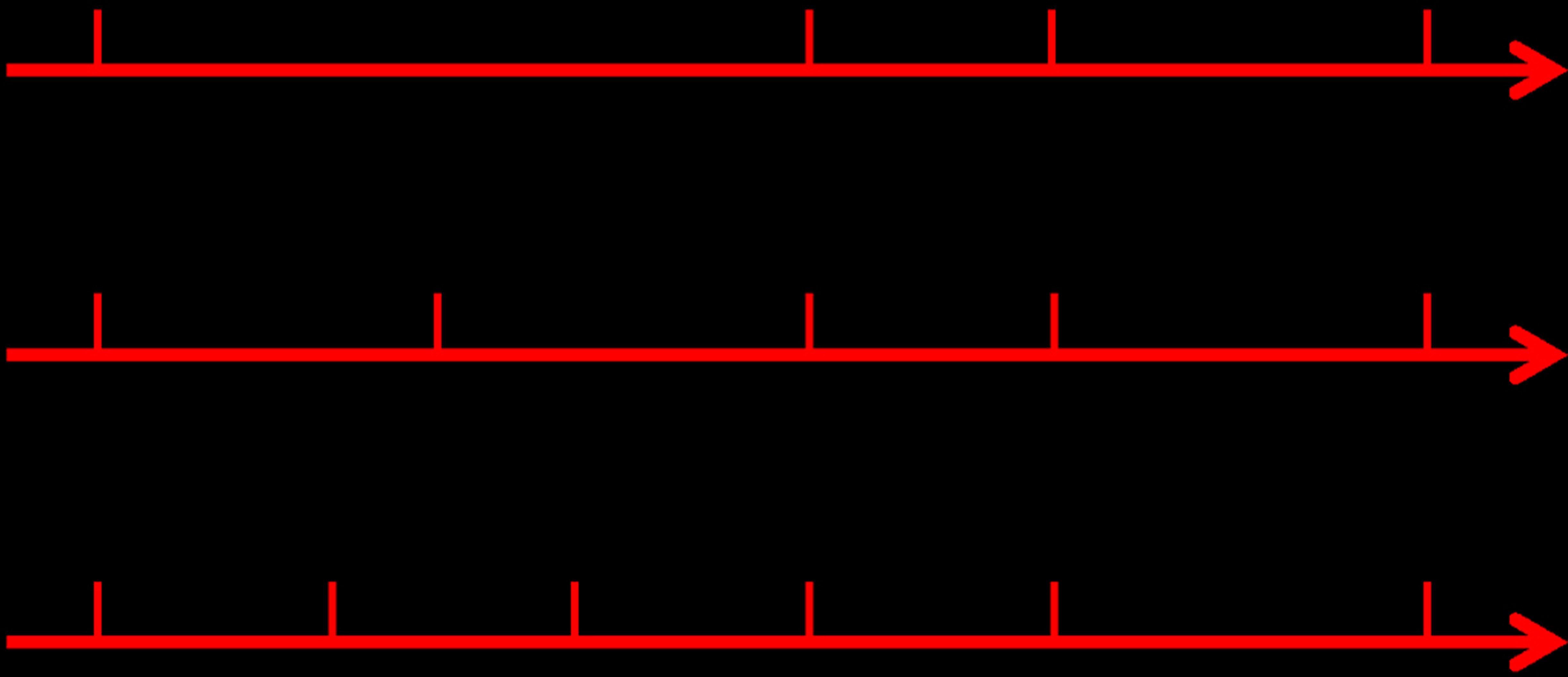

Supplement: Supplementary Figure S2 [file cddis2017371x2.tif]

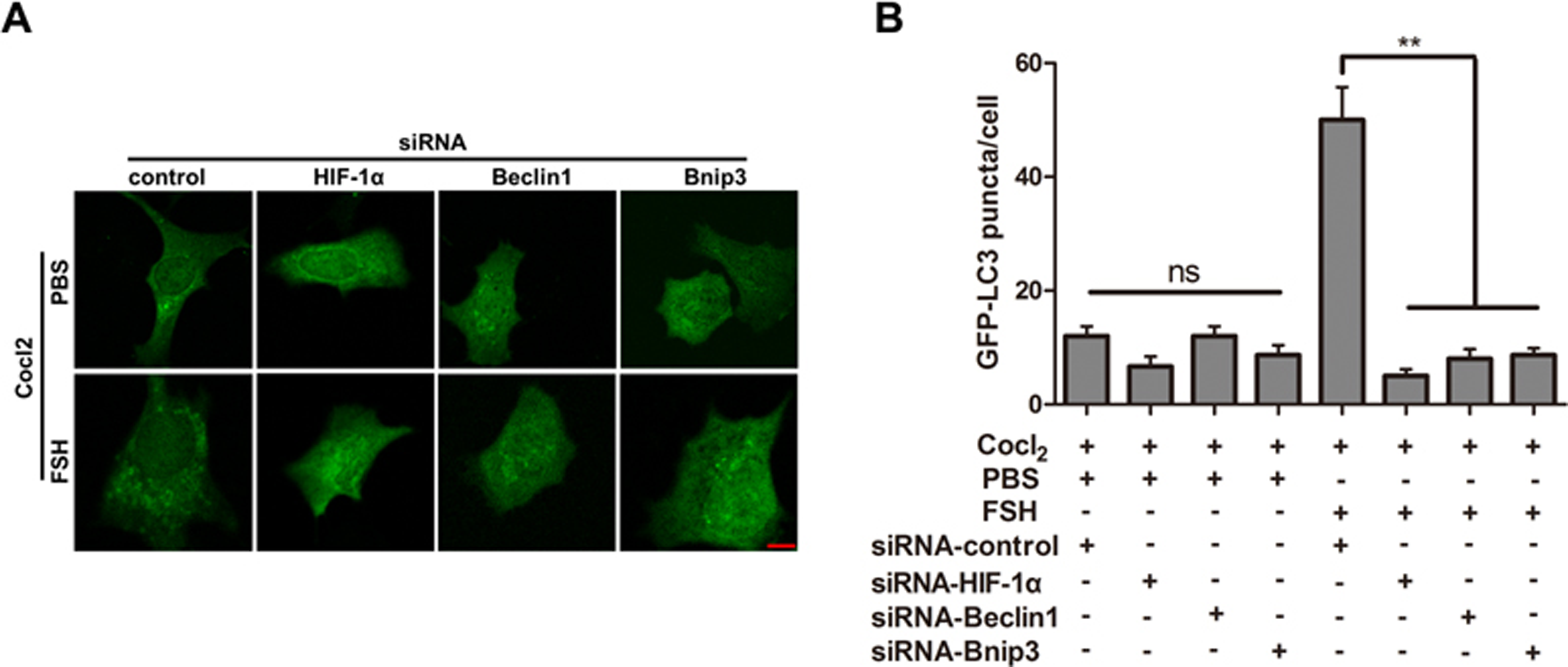

Supplement: Supplementary Figure S3 [file cddis2017371x3.tif]

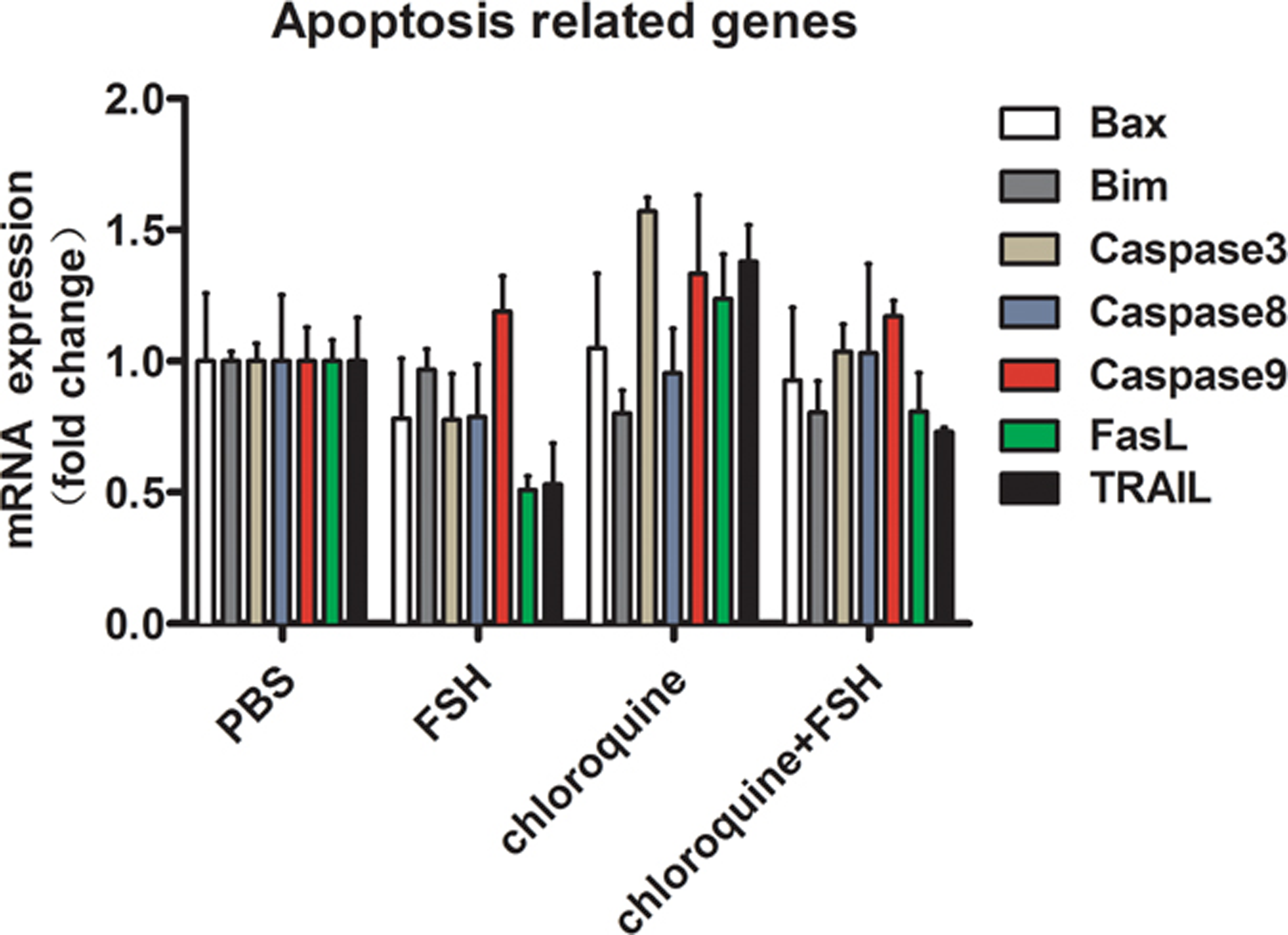

Supplement: Supplementary Figure S4 [file cddis2017371x4.tif]

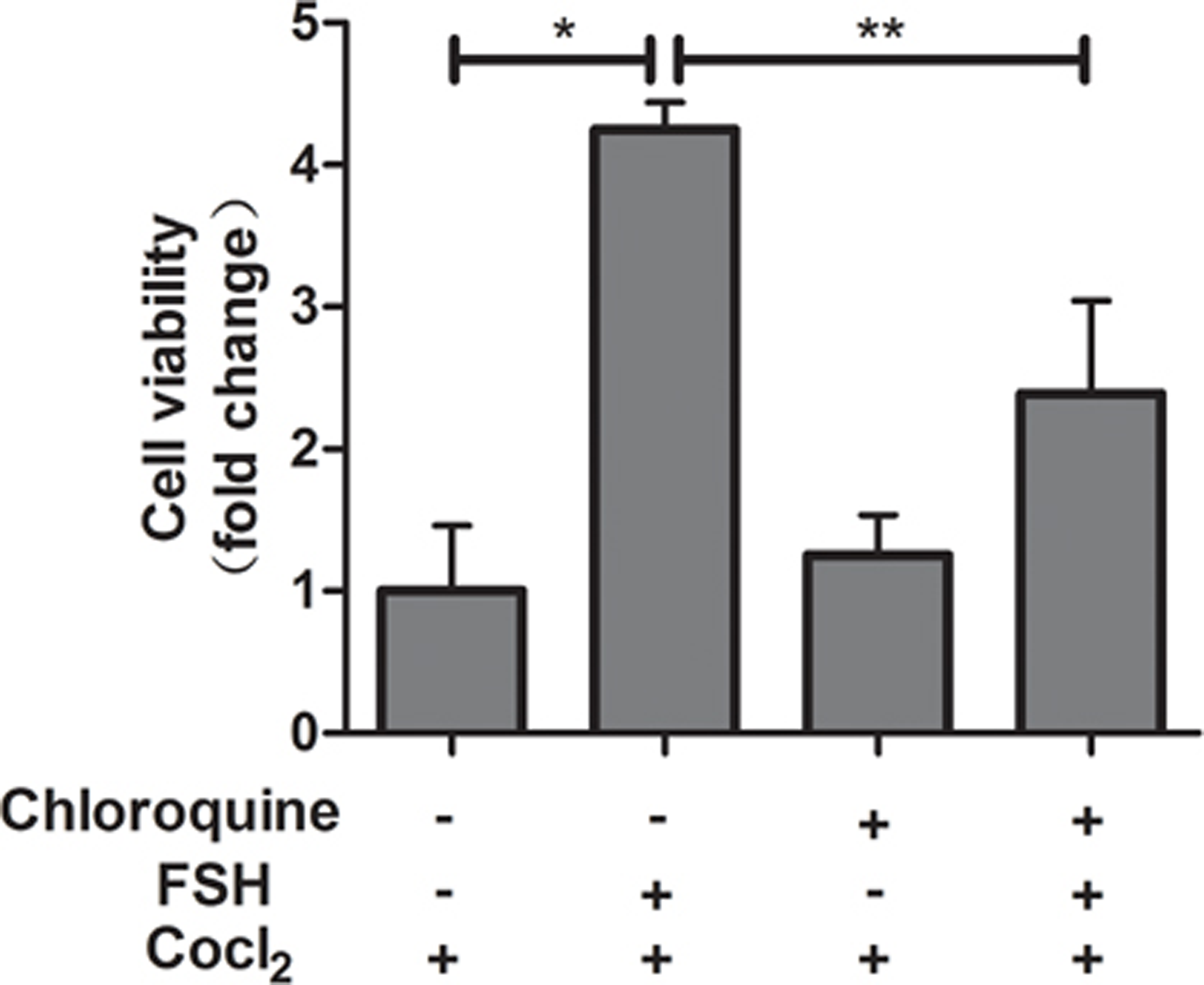

Supplement: Supplementary Figure S5 [file cddis2017371x5.tif]
