# Supplementary material for: Administration of follicle-stimulating hormone induces autophagy via upregulation of HIF-1α in mouse granulosa cells
Source: Cell Death Dis. 2017 Aug 17;8(8):e3001–. doi: 10.1038/cddis.2017.371 (PMC5596559; doi:10.1038/cddis.2017.371)
Supplement: Supplementary Figure legends [file cddis2017371x6.doc]

# Supplementary Files

# Supplementary Figure S1 The effect of FSH on MGCs viability. Mice were treated with FSH, cell viability was determined at 0, 1.5, 3, 6, 9, 12 h, and calculated relative to that of control group, cell viability of 0 h group was set at 100%. The data represent the means ± S.E; (n = 3).

# Supplementary Figure S2 The inhibitor experiment procedure. (A-C) To analysis the function of inhibitors Px-478 and Compound C on mouse ovaries under physiological conditions, we injected mice i.p. with inhibitors and then treated with FSH at a dose of 10 IU. (A). After mice treated with inhibitor for 24 h, FSH was then injected for an additional 3 or 9 h to promote MGC autophagy in MGCs. (B) Mice were treated with inhibitor twice at 24 h intervals, MGCs were collected after additional treatment of FSH for 3 h and 12 h. (C) Inhibitors were injected for 3 times at 24 h intervals, samples were collected after FSH treatment for another 3 h and 12 h.

# Supplementary Figure S3 Autophagy signaling in FSH treated and untreated HIF1-α, Beclin-1 and Bnip3 down-regulated granulosa cells. (A) After MGCs were transfected with siRNA and GFP-LC3 plasmid for 24 h, cells were treated with FSH or CoCl_2_, and GFP puncta were detected by immunofluorescence. Bar = 10 μm. (B) Quantitative analysis of the data in (A). The data are means ± S.E; (n = 3). *^**^* *p* < 0.01. ns, not significant.

**Supplementary Figure S4** Block of autophagy effects on MGC apoptosis. After treatment with or without chloroquine and FSH, the apoptosis related genes were determined by qPCR. The relative expression data were normalized to the amount of *GAPDH*. The data are means ± S.E; (n = 3).

**Supplementary Figure S5** FSH induced autophagy has an effect on cell viability. MGCs treated with Cocl_2_ and FSH were incubated with chloroquine at 24 h before the detection. Cell viability was determined by CCK8 assay. The viability of Cocl_2_ group was set at 100%. *^*^ p* < 0.05.The data represent the means ± S.E; (n = 3).
