# Supplementary material for: Administration of follicle-stimulating hormone induces autophagy via upregulation of HIF-1α in mouse granulosa cells
Source: Cell Death Dis. 2017 Aug 17;8(8):e3001–. doi: 10.1038/cddis.2017.371 (PMC5596559; doi:10.1038/cddis.2017.371)
Supplement: Supplementary Table S1 [file cddis2017371x7.doc]

**Supplemental table S1 Primer sequences used in real-time PCR**

| **Primer ID** | **Forward primer sequence (5’-3’)** | **Reverse primer sequence (5’-3’)** |
| --- | --- | --- |
| HIF-1α  AMPK  Catalase  MnSOD | TCA AGT CAG CAA CGT GGA AG  AAG CCG ACC CAA TGA CAT CA  TCTGGTGATATCGTGGGTGA  GAA CCT TGG ACT CCC ACA GA | TAT CGA GGC TGT GTC GAC TG  CTT CCT TCG TAC ACG CAA AT  CCT CGT TCA GGA TGT GGT TT  ATG GTG GGG GAC ATA TT |
| 3β-HSD | TGG ACA AAG TAT TCC GAC CAG A | GGC ACA CTT GCT TGA ACA CAG |
| INHα | CCT TTT GCT GTT GAC CCT ACG | AGG CAT CTA GGA ATA GAG CCT TC |
| stAR | ATG TTC CTC GCT ACG TTC AAG | CCC AGT GCT CTC CAG TTG AG |
| VEGF | GAG GTC AAG GCT TTT GAA GGC | CTG TCC TGG TAT TGA GGG TGG |
| CYP19A1 | ATG TTC TTG GAA ATG CTG AAC CC | AGG ACC TGG TAT TGA AGA CGA G |
| Bax | TGA AGA CAG GGG CCT TTT TG | AAT TCG CCG GAG ACA CTC G |
| Bim | TCG TCC ATC GAG GAT GAC TTC | TGC AGA GAG AGG ATA CTG TAG AC |
| Caspase3 | TGG TGA TGA AGG GGT CAT TTA TG | TTC GGC TTT CCA GTC AGA CTC |
| Caspase8 | TGC TTG GAC TAC ATC CCA CAC | TGC AGT CTA GGA AGT TGA CCA |
| Caspase9 | TCC TGG TAC ATC GAG ACC TTG | AAG TCC CTT TCG CAG AAA CAG |
| FasL | TCC GTG AGT TCA CCA ACC AAA | GGG GGT TCC CTG TTA AAT GGG |
| TRAIL | CCT GGA AAG CGA CTG AAC | ACC GAA AGT GTC TGT GGC |
| CDK1 | AGA AGG TAC TTA CGG TGT GGT | GAG AGA TTT CCC GAA TTG CAG T |
| CDK2 | CCT GCT TAT CAA TGC AGA GGG | TGC GGG TCA CCA TTT CAG C |
| cyclinA1 | TGA TGC TTG TCA AAT GCT CAG C | AGG TCC TCC TGT ACT GCT CAT |
| cyclinA2 | GCC TTC ACC ATT CAT GTG GAT | TTG CTG CGG GTA AAG AGA CAG |
| cyclinB1 | AAG GTG CCT GTG TGT GAA CC | GTC AGC CCC ATC ATC TGC G |
| cyclinB2 | GCC AAG AGC CAT GTG ACT ATC | CAG AGC TGG TAC TTT GGT GTT C |
| cyclinD1 | GCG TAC CCT GAC ACC AAT CTC | CTC CTC TTC GCA CTT CTG CTC |
| GAPDH | AGG TCG GTG TGA ACG GAT TTG | TGT AGA CCA TGT AGT TGA GGT CA |
